# Is Content Really King? An Objective Analysis of the Public's Response to Medical Videos on YouTube

**DOI:** 10.1371/journal.pone.0082469

**Published:** 2013-12-18

**Authors:** Tejas Desai, Afreen Shariff, Vibhu Dhingra, Deeba Minhas, Megan Eure, Mark Kats

**Affiliations:** 1 Division of Nephrology and Hypertension, East Carolina University, Greenville, North Carolina, United States of America; 2 Department of Internal Medicine, East Carolina University, Greenville, North Carolina, United States of America; 3 Department of Physiology, NC State University, Raleigh, North Carolina, United States of America; 4 Northeast Georgia Diagnostic Clinics, Gainseville, Georgia, United States of America; Tel Aviv University, Israel

## Abstract

Medical educators and patients are turning to YouTube to teach and learn about medical conditions. These videos are from authors whose credibility cannot be verified & are not peer reviewed. As a result, studies that have analyzed the educational content of YouTube have reported dismal results. These studies have been unable to exclude videos created by questionable sources and for non-educational purposes. We hypothesize that medical education YouTube videos, authored by credible sources, are of high educational value and appropriately suited to educate the public. Credible videos about cardiovascular diseases were identified using the Mayo Clinic's Center for Social Media Health network. Content in each video was assessed by the presence/absence of 7 factors. Each video was also evaluated for understandability using the Suitability Assessment of Materials (SAM). User engagement measurements were obtained for each video. A total of 607 videos (35 hours) were analyzed. Half of all videos contained 3 educational factors: treatment, screening, or prevention. There was no difference between the number of educational factors present & any user engagement measurement (p NS). SAM scores were higher in videos whose content discussed more educational factors (p<0.0001). However, none of the user engagement measurements correlated with higher SAM scores. Videos with greater educational content are more suitable for patient education but unable to engage users more than lower quality videos. It is unclear if the notion “content is king” applies to medical videos authored by credible organizations for the purposes of patient education on YouTube.

## Introduction

In recent years medical educators and patients are increasingly turning to YouTube to teach and learn about medical conditions, respectively. Although the creators of YouTube designed it for entertainment, rather than educational, purposes, users are producing and viewing videos about topics such as immunizations, prostate cancer, and kidney stones in greater numbers. [1]–[4]. Many of these videos are from authors/sources whose credibility cannot be verified [5]–[7]. An even greater number of videos are not peer reviewed [5]–[7]. Without a standardized peer-review process or a method by which credible sources can be identified, searches for videos on YouTube result in an ambiguous mix of educational- and entertainment-focused videos.

This dilution is evident in the published scientific literature. Since 2007, investigations have reported dismal results regarding the educational content of YouTube [5], [7]–[9]. As a result of these negative findings, research involving medical YouTube videos has plummeted 83% in one year [2]. However, prior investigations have not excluded videos created by questionable sources and for non-educational purposes [8]. Data from these studies do not portray an accurate picture of the educational quality of videos created by organizations focused on patient education. We hypothesized that credible healthcare organizations produce many highly educational and suitable medical videos and that the public significantly engages with these videos.

## Methods

### Identifying credible sources on YouTube

We defined credible videos and YouTube channels as those authored by organizations that have a publicly stated commitment towards patient education. We identified such organizations by querying the Social Media Health network at the Mayo Clinic Center for Social Media (http://network.socialmedia.mayoclinic.org). Healthcare organizations, located within United States and committed to patient education, comprise this network [10]–[12]. We identified the top four states that had the most member organizations with YouTube channels as of December 2012.

We selected videos focused on cardiac, vascular, or cardiovascular diseases from the YouTube channels of these organizations. These three disease entities account for the greatest composite cause of death in the United States [13], [14]. We identified videos containing content in any of these three subject areas through the video title and/or short video description. The authors ensured that all videos selected for analysis met these criteria. We collected publicly available data for each video, such as title, URL, author, and duration.

### Evaluating Educational Breadth of Videos

We did not evaluate the accuracy of each video because organizations within the Social Media Healthcare network had committed to providing accurate patient educational materials on YouTube. Rather, we focused on the type and breadth of content present in the videos. Since no validated scoring system existed to assess the content in a video, we devised a system in which we categorized educational content into one of 7 non-mutually exclusive domains: 1) epidemiology, 2) pathophysiology 3) screening, 4) diagnosis, 5) complications, 6) treatment/management, and 7) prevention. We obtained the definitions for each domain from the fourth edition of the American Heritage Dictionary of English Language or the American Heritage Medical Dictionary. Five authors (MK, AS, VD, DM, and ME) assessed the presence or absence of each domain within a particular video. These authors calculated a cumulative integer score between zero (no domains present) to seven (all domains present) for each video.

We performed an inter-rater agreement analysis by using videos that contained information about cardiac, vascular, or cardiovascular diseases, but did not meet the geographic inclusion criteria.

### Evaluating Suitability of Videos

We evaluated the degree to which the lay public could understand each video. Previous investigations have used the Suitability Assessment of Materials (SAM), a validated scoring system, to evaluate print-, audio-, and video-formatted patient education materials [15], [16]. We calculated the composite SAM score through the evaluation of 6 factors: 1) content, 2) literacy demand, 3) graphics, 4) layout and typography, 5) learning stimulation, and 6) cultural appropriateness [16]. We used the criteria within each factor to grade each video on an integer scale from zero (poor suitability) to two (superior suitability). The higher the SAM score (maximum score 42 for print material, 38 for video material), the easier the lay public could understand the material. We categorized videos as superior (70–100% of the maximum possible SAM score), adequate (40–69%), or inadequate (0–39%) based on the cumulative raw score [15], [16].

### Evaluating User Engagement

We recorded five measures of user engagement for each video: the number of 1) video views, 2) likes, 3) dislikes, 4) favorites, and 5) comments. We collected this data from February 6–8, 2013. We quantified the degree of independence for each metric against the other (e.g., Video views against Dislikes, Comments against Likes, etc.) because no such analysis was found in the medical literature. We defined videos with high user engagement as those with a large number of observations in any independent metric (e.g, large number of Views, Likes, Comments, etc.).

### The Optimal Video

We defined an optimal video as being both of great educational breadth and of superior suitability. We predefined a video of great educational breadth as one whose content was categorized in at least 4 educational domains. A highly suitable (superior) video, defined by the creators of the SAM scoring system, was any video that scored 70% or greater (raw score 27 or greater). Optimal videos exhibited both properties. We did not use the measures of engagement to identify optimal videos.

### Statistical Analyses

We considered video views, duration, and all user engagement metrics as continuous variables. We defined an independent user engagement metric as having a R^2^ between 0 and 0.55. We considered integer scores, obtained by assessing the educational breadth of content (educational domains), as ordinal variables. SAM scores were continuous variables. We recorded data on Google Spreadsheets and analyzed data with Microsoft Excel 2007 and JMP 10.1. Appropriate statistical tests are indicated within the body of the text and each table/figure. We defined statistical significance as results with a p value of less than 0.01.

This investigation was exempt from Institutional Review Board approval because it focused on publicly available educational material from organizations and not individual patients. We attempted as close adherence to STROBE guidelines as possible [17].

## Results

California, New York, Florida, and Texas had the most YouTube channels in the Mayo Clinic Center for Social Media Healthcare network. Of the 7,694 videos available from the network, 607 (8%) met inclusion criteria for further analyses (Table 1). We excluded one video (*Cardiologist speaks on the dangers of K2* at http://youtu.be/Owo-UU_u6Cc) from the analyses because it exhibited outlying tendencies (76,116 views; 13% of all views analyzed). The remaining 606 videos were of 34.9 hours duration (Table S1). We analyzed videos that accounted for 1.9% of the total video views. The median number of video views was 182 (IQR 63 and 518) and duration was 2 minutes (IQR 1.5 and 3.6 minutes). The Light's kappa score for inter-rater agreement was 0.76.

**Table 1:** Baseline characteristics.

	Hospitals with a YouTube Channel (No.)		Total (No.)		Cardiac, Vascular, or Cardiovascular (No.)	
State	No	Yes	Videos	Video Views	Videos	Video Views
California	23	19	1792	2,591,356	138 (8%)	141,317 (6%)
Florida	9	14	1727	1,526,870	250 (14%)	205,694 (13%)
New York	14	10	1883	23,345,937	100 (5%)	91,931 (0.4%)
Texas	12	13	2292	3,438,971	119 (5%)	146,096 (4%)
*Totals*	*58*	*56*	*7694*	*30,903,134*	*607 (8%)*	*585,038 (2%)*

**Figure 1:**
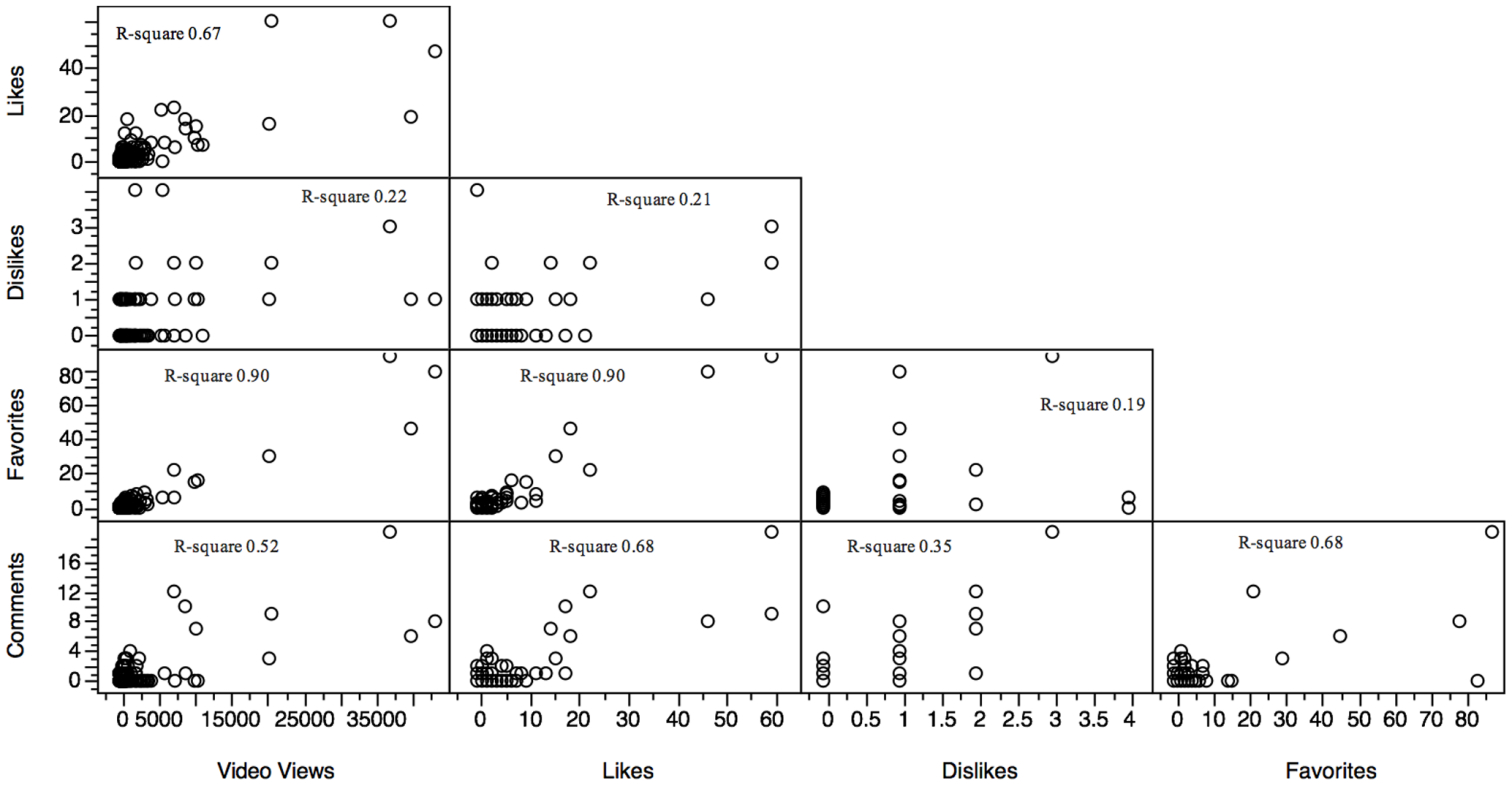
Scatterplot showing correlations between five user engagement measurements.

We measured user engagement using five metrics. Figure 1 shows the relationship between 3 of those metrics: Video views, Dislikes, and Comments. Both the number of Favorites and Likes exhibited a strong correlation with each other (R^2^ 0.90) and with the number of video views (R^2^ 0.90 and 0.67, respectively).

**Figure 2:**
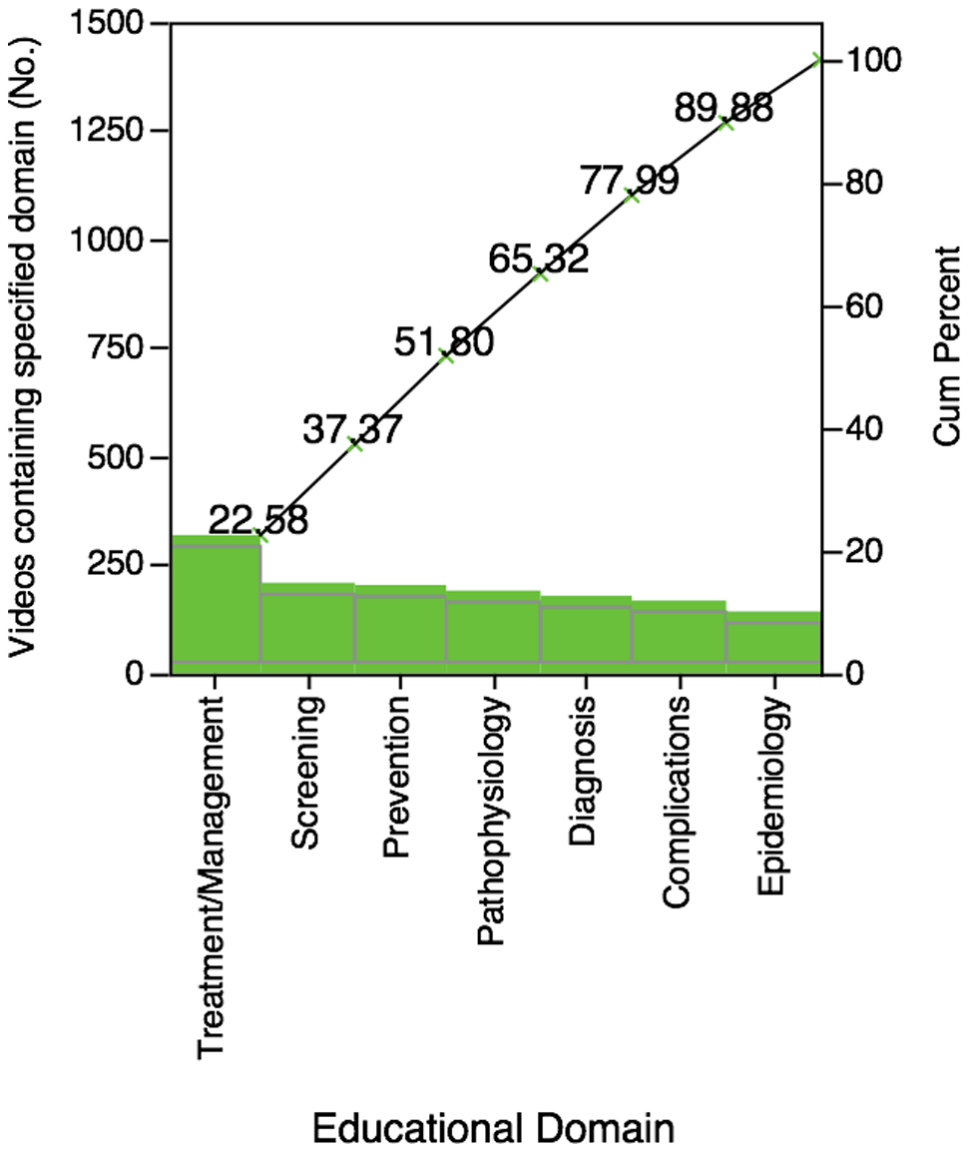
Pareto plot of educational domains present in YouTube videos.

Approximately 80% of all videos contained at least one educational domain (Table 2). The most frequently observed domain was Treatment/Management. Half of the videos contained at least 3 domains – treatment/management, screening, and prevention (Figure 2). A total of 449 videos had 3 or fewer domains and 157 had 4 or more domains (great educational breadth). While there was a statistical increase in the duration of videos with great breadth (6.8 versus 2.3 minutes, t-test p<0.0001), there was no difference in the number of video views (900 versus 826, p 0.6), dislikes (0.09 versus 0.08, p 0.6), or comments (0.22 versus 0.31, p 0.19).

**Table 2:** Breadth of educational content based on user engagement metrics and video duration.

		Number of Domains Present							
		0	1	2	3	4	5	6	7
N^a^ (%)		123 (20%)	122 (20%)	110 (18%)	93 (15%)	69 (11%)	46 (8%)	28 (5%)	15 (3%)
Video Views (No.)	Mean	557.20	380.32	1924.38	455.12	605.61	1325.29	849.68	1072.07
	Std Dev	1492.45	517.36	6928.36	962.84	1498.27	3343.78	2078.86	2406.10
* * ***ANOVA p 0.01***									
Video Duration (minutes)	Mean	1.96	2.21	2.27	3.05	3.29	5.14	8.04	25.37
	Std Dev	1.77	1.79	1.28	2.53	2.41	5.16	10.70	18.35
* * ***ANOVA p<0.0001***									
Likes (No.)	Mean	0.97	0.82	2.79	0.58	0.90	1.40	1.36	1.47
	Std Dev	2.90	2.22	9.66	1.10	1.51	2.85	3.28	3.58
* * ***ANOVA p 0.03***									
Dislikes (No.)	Mean	0.09	0.04	0.13	0.05	0.10	0.07	0.14	0.00
	Std Dev	0.53	0.24	0.46	0.23	0.30	0.25	0.45	0.00
* * ***ANOVA p 0.6***									
Favorites (No.)	Mean	0.71	0.62	3.99	0.57	0.35	1.59	0.14	0.17
	Std Dev	2.04	1.04	14.25	1.16	0.65	5.27	0.36	0.41
* * ***ANOVA p 0.01***									
Comments (No.)	Mean	0.18	0.12	0.78	0.19	0.21	0.18	0.35	0.11
	Std Dev	1.04	0.35	2.81	0.57	0.76	0.56	1.57	0.33
* * ***ANOVA p 0.05***									

^a^ Excludes 1 video.

The median SAM score was 24 (IQR 16 and 28). One-third of the videos were of superior suitability (203 videos) and 45% were adequate. Superior videos had a longer duration than adequate and inadequate videos (5.4 versus 2.8 versus 1.9 minutes, ANOVA p<0.0001). There were no statistical differences between any user engagement metric and the suitability of a video (Table 3).

**Table 3:** SAM scores based on user engagement metrics and video duration.

		Qualitative SAM Score		
		Inadequate^b^	Adequate^c^	Superior^d^
N^a^ (%)		128 (21%)	275 (45%)	203 (33%)
Video Views (No.)	Mean	558.8	1017.3	793.2
* * ***ANOVA p 0.4***	Std Dev	1475.2	3959.0	3231.0
Video Duration (minutes)	Mean	1.9	2.8	5.4
* * ***ANOVA p<0.0001***	Std Dev	1.8	2.1	9.1
Likes (No.)	Mean	0.9	1.6	1.1
***ANOVA p 0.3***	Std Dev	2.7	5.8	3.9
Dislikes (No.)	Mean	0.05	0.1	0.1
***ANOVA p 0.06***	Std Dev	0.4	0.4	0.2
Favorites (No.)	Mean	0.7	1.7	1.1
***ANOVA p 0.4***	Std Dev	1.9	7.8	6.6
Comments (No.)	Mean	0.2	0.4	0.2
* * ***ANOVA p 0.3***	Std Dev	1.0	1.8	0.8

^a^ Excludes 1 video.

–39%).^b^ Represents raw SAM score of 0 to 15 (0

–69%).^c^ Represents raw SAM score of 16 to 26 (40

% or greater).^d^ Represents raw SAM score of 27 to 38 (70

We predefined an “optimal” video as one with 4 or more educational domains and a superior suitability SAM score. In general, videos of great breadth had a higher SAM score than less educational videos (26.2 versus 19.5 respectively, ANOVA p<0.0001). However, there were far fewer optimal videos than all other videos combined (75 versus 531, Fisher's exact test p<0.0001). Although optimal videos were the longest videos of any type, they did not engage the user with greater frequency than any other video type (Table 4).

**Table 4:** Overall video quality based on user engagement and video duration.

		Overall Video Quality			
		Lower Educational Breadth, Inadequate, or Adequate	Great Educational Breadth Only^b^	Superior Only^c^	Optimal^d^
N^a^ (%)		320 (53%)	83 (14%)	128 (21%)	75 (12%)
Video Views (No.)	Mean	832.24	1024.84	810.80	763.47
	Std Dev	3502.49	2863.93	3891.62	1602.05
* * ***ANOVA p 0.96***					
Video Duration (minutes)	Mean	2.23	3.56	2.59	10.30
	Std Dev	1.86	2.44	1.99	13.41
* * ***ANOVA p<0.0001***					
Likes (No.)	Mean	1.36	1.32	1.13	1.03
	Std Dev	5.46	2.78	4.59	2.23
* * ***ANOVA p 0.93***					
Dislikes (No.)	Mean	0.09	0.15	0.06	0.03
	Std Dev	0.43	0.39	0.28	0.16
* * ***ANOVA p 0.22***					
Favorites (No.)	Mean	1.45	0.93	1.49	0.43
	Std Dev	7.02	3.91	7.96	1.34
* * ***ANOVA p 0.74***					
Comments (No.)	Mean	0.35	0.29	0.22	0.12
	Std Dev	1.74	1.07	0.87	0.47
* * ***ANOVA p 0.67***					

^a^ Excludes one video.

^b^ Represents videos containing 4 or more educational domains.

% or greater).^c^ Represents videos with a raw SAM score of 27 or greater (70

^d^ Represents videos containing b and c.

We compared each educational domain against Video Views, Dislikes, and Comments to identify any domain that was disproportionately represented in the videos with high user engagement. Table 5 shows the individual parameter estimates for the 7 logistic regression models – one for each domain. The range of AUCs was 0.48 to 0.58, suggesting that videos with high user engagement could not be characterized as containing a specific educational domain. Moreover, videos with high user engagement did not have a different video duration than videos with lower engagement (R^2^ 0.009).

**Table 5:** Logistic regression and receiver operator characteristics based on user engagement.

	Parameter Estimates (*β*)<				Receiver Operator Characteristic (ROC)
Outcome	Video Views	Dislikes	Comments	Intercept	Area Under Curve (AUC)
Epidemiology	2.87*10^−5^	−0.34	0.16	1.25*	0.54
Pathophysiology	−4.3*10^−5^	−0.55	0.059	0.75*	0.56
Screening	7.19*10^−7^	−0.37	0.13	0.66*	0.48
Diagnosis	−3.5*10^−5^	−0.37	0.10	0.82*	0.58
Treatment/Management	−7.22*10^−5^	−0.098	0.10	−0.094	0.55
Complications	−2.7*10^−5^	−0.18	0.03	0.97*	0.55
Prevention	2.1*10^−5^	−0.26	0.06	0.68*	0.53

*p<0.01.

## Discussion

Authoritative/credible healthcare organizations produce few highly educational and/or suitable medical videos. Additionally, the general public does not engage with those videos that are 1) highly educational, 2) of superior suitability, or 3) optimal. As a result, we were unable to reject the null hypothesis.

We based our hypothesis on the premise that credible organizations would author a large number of optimal videos and that the public would engage with those optimal videos. By eliminating entertainment videos that could have been disguised as educational, we believed that this investigation would uncover the true public response towards medically focused YouTube videos. The Social Media Healthcare network allowed us to capture only those YouTube videos that fulfilled our requirement for accuracy and credibility. Nevertheless, only 27% of videos were of great educational breadth (having 4 educational domains or greater). This percentage is similar to that found by other investigators, including those that have studied videos about prostate cancer (27%) [7]. Overall, previous investigations have uncovered that less than half of videos analyzed met any criteria to be considered as having great educational breadth [9], [18], [19]. In contrast, use of the SAM scoring system revealed 33% of videos as superior. This frequency is similar to those seen in comparable investigations. In one study, 11% of heart failure videos were considered superior, while no greater than 55% of videos pertaining to cardiac catheterization were superior [20], [21].

The public had an unfavorable response to optimal videos. Despite fewer optimal videos, the public did not view these videos or provide comments with greater frequency. Previous investigations regarding cardiopulmonary resuscitation or influenza vaccinations showed a similar disconnect between video quality and user engagement [7], [8], [22]. In extreme cases, user engagement was greater for inaccurate videos [23], [24]. The lack of correlation between public response and video quality should be a concern for any medical provider or organization. Since the 1990s it has been known that media images “strongly shape the public's [understanding] about medicine, illness, and death” [25]. Since that time, many investigations have revealed incorrect or misleading images by the media. Decades later, this finding persists. It appears that neither educational breadth nor suitability nor authorship can affect user engagement.

Surprisingly, the greatest strength of this investigation did not result in findings that were dissimilar to those of previous investigations. The focus on videos produced by 1) reputable organizations that have 2) committed to publishing accurate medical videos about 3) the leading composite causes of death in the United States did not garner a better public response. Using the Social Media Healthcare network to “drill down” onto credible and accurate medical videos failed to uncover a more positive user experience. Our second strength involves the sample size. Previous investigations analyzed videos numbering between 29 to 199 and a total video duration between 216 to 765 minutes [5], [7]–[9], [18], [19], [22]–[24], [26]. Our investigation analyzed 607 videos for a total video duration of 2,094 minutes, providing a sample size unmatched in prior studies. Finally, few studies have objectively analyzed educational content or suitability and concomitantly avoided grading scales such as “fair”, “poor”, “useful”, or “not useful” [7], [9], [18], [20], [27], [28]. This investigation utilized an objective methodology to evaluate videos for educational content and suitability.

## Conclusions

Is content really king? Media experts preach this concept as a key to successfully communicating with the public. In recent years, however, investigations such as ours have yielded data that call this notion into question. Content and quality may be key ingredients for entertainment-focused organizations. When the goal of content is to educate the public, however, healthcare organizations do not produce many optimal videos. When an organization authors an optimal video, the general public is no more likely to engage with it than with less optimal videos. Indeed we cannot predict how the public will engage with a video based on 1) educational breadth, 2) SAM score, and/or 3) publicly available metadata. This uncertainty poses a major challenge to healthcare organizations. Neither a commitment to creating accurate medical videos nor creating videos with great educational breadth or high suitability results in a positive user experience. Thus, whether the content is delivered through medical-based television shows (e.g., *Rescue 911*) or YouTube videos authored by reputable healthcare organizations, it is unclear if “content is king” in medical education videos [25].

## Supporting Information

Table S1 (PDF)
