# Supplementary material for: Is Content Really King? An Objective Analysis of the Public's Response to Medical Videos on YouTube
Source: PLoS One. 2013 Dec 18;8(12):e82469. doi: 10.1371/journal.pone.0082469 (PMC3867348; doi:10.1371/journal.pone.0082469)
Supplement: Table S1 — (PDF) [file pone.0082469.s001.pdf]

Video Title	URL
Symptoms of Aortic Dissection	http://youtu.be/OSZMATDWMd8
TAVR (Transcatheter Aortic Valve Replacement) - Dr.Lewis Britton	http://youtu.be/LfDcqd-csMQ
TAVR (Transcatheter Aortic Valve Replacement) - Dr. Edward V. Bennett	http://youtu.be/qqHkxiMm44Y
TAVR (Transcatheter Aortic Valve Replacement) - Dr. Manish Mehta	http://youtu.be/FYQiGck7ta8
Bennett and Patient - First Robotic Mitral Valve	http://youtu.be/6S584JqLL2k
Chloe: A Survivor Story	http://youtu.be/ns23IdTdOZ8
First Long Island Cryoablation Procedure Performed at NSLIJ's Southside Hospital	http://youtu.be/e6CaGX0pp7Q
Healing Heart & Bone	http://youtu.be/tWHe5rFtP7g
Rare Stroke Recovery: Rachel's Story of Rehabilitation and How She Helps Others	http://youtu.be/lhC6ne0T_g0
Southside Hospital Welcomes First Lariat Heart Patient on Long Island	http://youtu.be/sd83c2zyl_Q
Advances and Techniques of Minimally Invasive Heart Surgery	http://youtu.be/xGJRO47cJnQ
Patrick's Story: Living an Active Life Despite Being Born with Pulmonary Valve Stenosis	http://youtu.be/fTeRdsHyFYY
New Cardiac Units at LIJ Designed for Advanced Care, Comfort	http://youtu.be/obnZ7ZAR0RM
Heart Health: What is Target Heart Rate?	http://youtu.be/EX8vr_sAkR8
New Nonsurgical Heart Valve Replacement Offered at LIJ	http://youtu.be/ea7ub0z5I1c
Take Charge of Your Heart Health: Know Your Numbers	http://youtu.be/T5xY6AdRyfY
Broken-heart syndrome	http://youtu.be/xayolotjAv4
UC Davis Health System presents "What Do You See?" - Stereotaxis Heart Procedure	http://youtu.be/bXKqL_lzfAc
Arabella and Adriana Cipponeri - Fetal Heart Surgery Saves Twins	http://youtu.be/kcFiTViUBec
KPIX on High Blood Pressure in Kids	http://youtu.be/iNW9sb71luY
Children's Hospital Oakland -- KTVU Reports on Statins for Children	http://youtu.be/-nFzzPArV7E
Children's Hospital Oakland - Pediatric Cardiology, pt. 4	http://youtu.be/mGlge39ZeXk
Children's Hospital Oakland - Pediatric Cardiology, pt. 3	http://youtu.be/K5CsBAksq0w
Children's Hospital Oakland - Pediatric Cardiology, pt. 2	http://youtu.be/y7AjlR7kDow
Children's Hospital Oakland - Pediatric Cardiology, pt. 1	http://youtu.be/sy_zEu1HhTA
Children's Hospital Oakland - Dr. Kathleen Newkumet	http://youtu.be/LL7DQw0e2js
Children's Hospital Oakland - KNTV Reports on Amazing Hockey-Playing Cardiology Patient	http://youtu.be/b31PfyemoYQ
Children's Hospital Oakland - Cutting Edge Research	http://youtu.be/jFcWiRtTVX0
Children's Hospital Oakland - Fetal Echo Program	http://youtu.be/-Ajc5YJO5uI
Eat Heartily: Recipes for Heart-Healthy Eating	http://youtu.be/1XQEVyoJEbA

A Better Heart Without Surgery — Stephanie's Valve Replacement Story	http://youtu.be/JPKh299sqq4
What do I need to know about Atrial Fibrillation?	http://youtu.be/aNWILnEfChg
Albany Med Heart Care - Arthur	http://youtu.be/W7d8T2bD8zQ
Heart Care at Albany Med - Ernest	http://youtu.be/Zb2ZtfcS49o
Heart Care at Albany Med	http://youtu.be/LfzPdG5M1E0
LVAD Patient Kurt Maschewski	http://youtu.be/IY7ysjYFz9g
LVAD Patient John Scott	http://youtu.be/q4M7i5djGzc
LVAD Patient David Morey	http://youtu.be/Ynpv8iesSnw
LVAD Patient Daniel Demmer	http://youtu.be/wQXN-NebB2A
TAVR (Transcatheter Aortic Valve Replacement) - Dr. Augustin DeLago	http://youtu.be/eQ-BpF8v88g
Chuck Greenberg of Habitat for Humanity, Describes his Stroke and Recovery	http://youtu.be/P3l4S4cIgtA
SJHS 4 Your Health Featuring Dr. Mohammad Otahbachi	http://youtu.be/Qdh_VdGLFCw
SJHS 4 Your Health Featuring Dr. Andrew D. Michaels	http://youtu.be/y6kHAzjCZ7U
SJHS 4 Your Health Featuring Dr. Peter Chang-Sing	http://youtu.be/IKOD3RQDhyI
SJHS 4 Your Health Featuring Dr. Christopher Gibson	http://youtu.be/kW9JE38f4J0
SJHS 4 Your Health Featuring Dr. Jeffery Yablon	http://youtu.be/TVtA6s-jzsI
SJHS 4 Your Health Featuring Dr. Christopher Celio	http://youtu.be/naGVGkDYdK8
American Heart Month	http://youtu.be/zvdUh2HbUx4
Bone marrow transplants: Genetics linked to heart failure after chemotherapy | City of Hope	http://youtu.be/XUnooaqEzG4
Mercy's Day of Dance, 2012	http://youtu.be/oGT47uzBVQQ
Patient Testimonial - da Vinci Cardiac Surgery	http://youtu.be/fZ7OgV_x-7E
KCRA 3 - Pregnancy & Pacemakers	http://youtu.be/tOwihoThZ68
KCRA 3, KTXL-FOX 40, News10 - Cardiac Patient Reunion	http://youtu.be/EamyUR_ySpo
KOVR-CBS 13 - Soccer Player Gets CPR	http://youtu.be/81N1XEmxbrg
Mercy's Day of Dance, 2012	http://youtu.be/opB1mnlI3kY
KOVR - Cormatrix	http://youtu.be/4yFCTu42zV0
Frank Slachman, MD - Robotic-Assisted Cardiac Surgery	http://youtu.be/Y2-KSYZ1Bi0
Allen Morris, MD - Robotic-Assisted Cardiac Surgery	http://youtu.be/Oye0q1HKp2g
KCRA / KOVR - Life Saved with CPR	http://youtu.be/K9_l_UwgkbM
Mercy's TeleStroke Program	http://youtu.be/VdKf3RWwGUk
Good Day Sacramento - Cardiologist Kathy Glatter, MD	http://youtu.be/QHyWTR-2xOc

FOX 40 News - Cardiologist Jonathan Hemphill, MD	http://youtu.be/qN0Wgjc3Hro
KMAX TV - Cardiologist Michael Chang, MD	http://youtu.be/F0iufToDhMI
Padraig O'Neill, MD - Mercy Heart & Vascular Institute	http://youtu.be/3ikFckPGqoc
Karanjit Singh, MD - Mercy Heart & Vascular Institute	http://youtu.be/tD_LuVOBNYU
Joseph Kozina, MD - Mercy Heart & Vascular Institute	http://youtu.be/AtNlMDAvg7Y
Michael Chang, MD - Mercy Heart & Vascular Institute	http://youtu.be/JY1UjQ0Mrk0
Walt Marquardt, MD - Mercy Heart & Vascular Institute	http://youtu.be/d_X1G7_sLaQ
Arvin Arthur, MD - Mercy Heart & Vascular Institute	http://youtu.be/Hy0ise3AKZ8
Kathy Glatter, MD - Heart disease IS a women's disease	http://youtu.be/eaIbIrh4wAY
Stroke Patient Inspires Himself and Others	http://youtu.be/Wr7qA0dSHkw
Stayin' Alive with Sutter Heart & Vascular Institute	http://youtu.be/quEGgsF4aCI
Atrial Fibrillation (AFIB) Minimally Invasive Treatment	http://youtu.be/eCgM1eB7ezk
Ventricular Assist Device (VAD) for Heart Failure	http://youtu.be/o7eHdzsWe4Y
Impella for Heart Failure or Heart Transplant	http://youtu.be/6ZseKBVJECY
Trans-apical Approach to TAVR	http://youtu.be/xZuXlgK3beU
Actual Transcatheter Aortic Valve Replacement (TAVR)	http://youtu.be/egwGwDi6pak
Pseudoaneurysm avi	http://youtu.be/N55iVqvbTVc
SFA Embolic event avi	http://youtu.be/desMYeXg0oY
SFA with stent avi	http://youtu.be/DrfKLjnRoNw
Carotid bifurcation avi	http://youtu.be/w7_X_n4p1xo
Aortic aneurysm Live WEB	http://youtu.be/zgjOkAx4aVE
transfemoral	http://youtu.be/UOTnyoIGCTg
Dr. Aidan Raney discusses heart surgery on Healthy OC	http://youtu.be/ZlfyMecFJfo
Transcatheter Aortic Valve Implantation	http://youtu.be/_eiI6S62zOs
Dr. David Brown and Dr. Michael Brant Zawadzki- Treating Stroke	http://youtu.be/1kxwj50egh0
Endovascular Procedures at Hoag	http://youtu.be/bVoF-jVwyJM
What to Expect Before & After an Endovascular Procedure at Hoag	http://youtu.be/vF-Hp6DXy8Q
Hoag Valve Center	http://youtu.be/1og0UWw8tg0
Dr. Mahmoud Eslami discusses Peripheral Vascular Disease	http://youtu.be/zJq91djvtvI
Dr. Michael Panutich discusses Electrophysiology	http://youtu.be/Tr8tEA0L90M
Dr. Douglas Zusman discusses Heart Valve Disease	http://youtu.be/s5CwunwLZfk

Dr. Bahram Eslami discusses Cholesterol	http://youtu.be/vCTfXkGlu9k
Dr. Aidan Raney discusses heart disease	http://youtu.be/T9EnKNDorNE
Dr. Subbarao Myla discusses preventing a heart attack	http://youtu.be/U-97GXuolss
Dr. Rajeesh Banker Discusses Arrhythmia and Electrophysiology	http://youtu.be/i5kkWM_FbZ4
Dr. Aidan Raney Discusses Heart Valve Surgery	http://youtu.be/pi3cd7fL1Mk
Dr. Zusman Discusses Atrial Fibrillation	http://youtu.be/lIyCLKPg27M
Dr. Haskell Discusses Coronary Stenting	http://youtu.be/Xczi4pK241c
Dr. Bahram Eslami Discusses High Blood Pressure	http://youtu.be/04bR2CzBcBQ
Dr. Michael Panutich Discusses Device Therapy	http://youtu.be/XeD6o8Efsiw
Dr. Jacques Kpodonu discusses Aortic Aneurysm	http://youtu.be/zs6LD5HwGV0
Dr. Bahram Eslami Discusses Preventing a Heart Attack	http://youtu.be/Qo9jL0xf--k
PS Animation Loop H264	http://youtu.be/h8Or-UZgNUw
Dr. David Brown Discusses Hoag's Stroke Program on Healthy OC	http://youtu.be/MekGFvw_lTU
Dr. Subbarao Myla and Dr. Jacques Kpodonu Discuss Peripheral Vascular Disease	http://youtu.be/gYD2yDcF0dc
Dr. Pravin Shah Discusses Heart Valve Disease	http://youtu.be/MG9kueVzY9E
Dr. Jacques Kpodonu Discusses Hybrid Heart Surgery	http://youtu.be/8xhK6nqVmyU
Dr. Subbarao Myla Discuses Carotid Stenting	http://youtu.be/pnahovjnhMk
Dr. Michael Panutich Discusses Electrophysiology and Heart Devices	http://youtu.be/28xPiSM2nHE
Dr. Richard Haskell Discusses Cardiac Arrest	http://youtu.be/ewsk-wHrqVM
We Lead The Way in Heart Care	http://youtu.be/CdaROSO5d9Y
Kaiser Permanente Colorado - 2012 Hypertension Control Champion	http://youtu.be/Ho0OybnkOhA
Healthy, Latin-inspired Dishes for the New Kaiser Permanente Westside Medical Center	http://youtu.be/e5CWchPCHIk
Kaiser Permanente Member Thanks Cardiologist for Saving her Life	http://youtu.be/XBCYP6RVEbQ
Yong Shin, MD, Shares Heart Health Advice	http://youtu.be/SJ8y0npAP1A
One on One with Ralph Brindis: Hypertension & Heart Health	http://youtu.be/Wwm-hdEjALg
One on One with Elijah Saunders on Hypertension & Heart Health	http://youtu.be/yB6LTYF7oxA
One on One with Janet Wright: Heart Health	http://youtu.be/hLPi1i2abeg
Hypertension Event at the Kaiser Permanente Center for Total Health	http://youtu.be/JotNNKWMni4
Kaiser Permanente Santa Clara Medical Center Heart Transplant Recipients Celebrate the Hol	http://youtu.be/D3HuLKkX2FQ
Cardiovascular Surgery Keeps Farmer in His Field	http://youtu.be/SER2Mk2npSY
Raising HDL Levels in South Asians	http://youtu.be/5dcz8j9Wlis

Lowering Your Triglycerides for South Asians	http://youtu.be/92h28uqBf0I
Understanding Cholesterol in South Asians	http://youtu.be/b0CHVKbp6Ms
PAMF Research on Cardiovascular Disease in South Asians	http://youtu.be/92EtUhCVIAo
Heart Valve Replacement Surgery: No Open Chest	http://youtu.be/A5-ZRxbfUD0
Less Invasive Mitral Valve Regurgitation Repair	http://youtu.be/VFGcBbTOUnE
Heart Forum 2012: Curative Procedures for Atrial Fibrillation	http://youtu.be/d9iSHlL0oP0
Heart Forum 2012: Transcatheter Valve Therapies	http://youtu.be/S62tdF_6RsA
Cardiovascular Advances From Head to Toe	http://youtu.be/iAkRRdpo6o4
Cardiovascular Risk Reduction: How to Beat the Odds	http://youtu.be/T08zQ7Mq3Hk
Ethnic Health Differences and Cardiovascular Risk	http://youtu.be/mzFVo3SaWCA
Gender and Cardiovascular Disease--Men and Women Present Differently	http://youtu.be/KhFLBxf1mEE
Women and Heart Disease	http://youtu.be/gg6CtCLcQak
The Fogarty Institute for Innovation Partners with Cardiologists	http://youtu.be/iTh-Fy49DNc
Critical Heart Attack Distinctions for Women and Southeast Asians	http://youtu.be/basfSz_VGOk
El Camino Hospital PulsePoint App	http://youtu.be/e0nvK3QVajQ
The Atrial Fibrillation Connection to Stroke & Heart Failure	http://youtu.be/EGNn36ztZaw
Innovations in Robotic Surgery for Heart and Lung - El Camino Innovates	http://youtu.be/rJnXMcGwsMI
Evolving Surgical Management of Atrial Fibrillation	http://youtu.be/VLGmyWtS6Rg
Peripheral Vascular Disease: Latest Trends and Techniques	http://youtu.be/Km2jKgP-4As
Transcatheter Aortic Valve Implementation: Invite Me to Your 100th Birthday!	http://youtu.be/i1KjOTWA-s8
Hybrid Open and Endovascular Treatment of Aortic Aneurysms	http://youtu.be/nA7InuJDmTM
Percutaneous Mitral Valve Repair: 2011 Update	http://youtu.be/F8ocidfAh48
Medical Innovations in Treating Heart Disease	http://youtu.be/ImMTiO17nDM
Higher Risk of Heart Disease for South Asians	http://youtu.be/SzOQDHksmcM
Fixing Irregular Heartbeats - El Camino Innovates	http://youtu.be/BLPQTEyvr1s
Peripheral Artery Disease Warning Signs	http://youtu.be/O_MEXGT4FjU
Stroke Prevention: New Carotid Artery Treatment	http://youtu.be/If2OCUzyaTU
New Heart Attack EKG Technology Begins with 911	http://youtu.be/_lJxsFZUWbY
80% With Erectile Dysfunction Get Heart Disease	http://youtu.be/VafxRZQnVUw
Knowing Stroke Symptoms Saves Mother and Child	http://youtu.be/jVAymyOMQlM
Reduce Your Stroke Risk: ABCs of Stroke Prevention	http://youtu.be/REKZ5paebXM

Look for the Gold Seal of Approval for Stroke Care	http://youtu.be/45zHYTvwEXU
How to Survive a Stroke: Know Stroke Symptoms	http://youtu.be/s-aDFZ-HPGw
Is Your Chest Pain a Heart Attack?	http://youtu.be/k6Pj-K97bLs
Fast Action for Heart Attack Saves a Life	http://youtu.be/-A32MAMg9kU
Medical Update: Stroke Signs, Aneurysm Indications	http://youtu.be/0pf07XhX6nI
Peripheral Artery Disease Risks & Warning Signs	http://youtu.be/4RnRiCc1yQk
How to Prevent Cardiovascular Disease Damage	http://youtu.be/p3QoNiUa5Zc
Leading Irregular Heartbeat (Arrhythmia) Treatments	http://youtu.be/l0HokCrLeG0
El Camino Hospital: Electrophysiology Services	http://youtu.be/IMo0i_-G6-k
Stroke & Neurological care at Pomerado Hospital	http://youtu.be/8CUuz7bUrqY
Lucky to be Alive: Southside Hospital Saves Crash Victim Suffering from an Aortic Transection	http://youtu.be/UOCw6H0eO-M
North Shore-LIJ Kicks Off Heart Health Month with Roundtable Discussion	http://youtu.be/PH5R0JE8Vus
Heart Health: What Women Should Know	http://youtu.be/h7KKvI0Ndps
LIJ Doctors Save Girl Suffering from Pulmonary Embolism	http://youtu.be/zYUonAZcZeA
Mini Medical School 2011 What is Heart Disease and What are the Risk Factors	http://youtu.be/7UkxYhCiC3s
Mini Medical School 2011 What Can You Do To Treat Heart Disease	http://youtu.be/WikWKcPqrMk
Mini Medical School 2011 How is Heart Disease Diagnosed	http://youtu.be/0lJWbrjyyt4
Mini Medical School 2011 Don't Go Breaking My Heart	http://youtu.be/81Migu0hkDA
Medical Update: Stroke - A Heart Attack of the Brain	http://youtu.be/Eu22_EX8GMA
Medical Update: Stroke Intervention	http://youtu.be/jXDWCHUlNUY
Lifestyle Changes Help Stroke Survivor Stay Healthy Four Years Later	http://youtu.be/-pc2lp4mMM0
Heart Failure	http://youtu.be/zGeqtxWZd3k
Your Heart's Electrical Current	http://youtu.be/tlaG5Qz60wU
How 2 Choose a Cardiologist	http://youtu.be/Y4ZYV-9UnxM
Coronary Artery Bypass Graft Surgery - What Is It?	http://youtu.be/QCeLEa95cEs
Angioplasty: What is It ?	http://youtu.be/fL3Aak_PI-I
Triple Bypass - Hillary's Story	http://youtu.be/ln9GkoHvqyc
Hypertension	http://youtu.be/hr5HFxLOsIs
St. Joseph's Electrophysiology Lab - Celebrating 15 Years	http://youtu.be/V_tENu-jHak
St. Joseph's EP Lab - Celebrating 15 Years	http://youtu.be/bswZX4sKzbM
Resolute Integrity Drug-Eluting Stent (DES)	http://youtu.be/gqIOATLdnLs

Transcatheter Aortic Valve Replacement (TAVR)	http://youtu.be/jtSZZWfSr4Q
St. Joseph's Hospital saves your life from Heart Attack	http://youtu.be/L8IH03b1zZg
Transradial Cardiac Catheterization Explained	http://youtu.be/apsZSVlfuig
Crouse Pediatric Cardiologist Craig Byrum, MD, Explains Procedure to Fix Child's Heart	http://youtu.be/0dpB081HpNc
Stroke Awareness Month May 2012	http://youtu.be/UNS-y4hbRsw
Having a Heart Attack? Take Me to Crouse	http://youtu.be/nOKcUoqBqbo
Crouse Hospital Cardiologist Matthew Gorman, MD, Talks About Heart Disease Prevention	http://youtu.be/UMeHoy0zncI
Crouse Dietitian Offers Advice on New Salt Intake Guidelines	http://youtu.be/OkfN2X5R8nY
Crouse Hospital Uses New LIFENET Technology for Heart Attack Patients	http://youtu.be/6Soqcktk1tc
Crouse Hospital Offers New Procedure for Carotid Artery Disease	http://youtu.be/hOhdXyaA2f0
Tomatoes for Stroke Prevention?	http://youtu.be/GZ81XamEqZQ
Health Benefits of Chocolate	http://youtu.be/LcAGqMHVh5Y
Managing Triglyceride Levels	http://youtu.be/x3Emxp6DxQk
Heart Health Cookoff: Kids With Heart Disease	http://youtu.be/KPrpEmGw-_s
High Blood Pressure During Pregnancy	http://youtu.be/_gyjCT0jP8Y
Blood Pressure During Pregnancy	http://youtu.be/IgZlQBLHRr0
Treating High Cholesterol	http://youtu.be/mMOZ53MxUqQ
Heart Healthy Diet	http://youtu.be/jaXfV_Q4Rbc
Prevalence of Heart Disease	http://youtu.be/JxD8YUO7r78
Heart Attack Warning Signs	http://youtu.be/O8f6g7YHSmg
Obesity and Heart Disease	http://youtu.be/D1FyN3sA5nk
Heart Disease Definition	http://youtu.be/dhrlConGzcY
Heart Disease Treatment	http://youtu.be/OFZpu11VIVE
Heart Disease Prevention	http://youtu.be/9vFEBb2ZU7c
Good and Bad Cholesterol	http://youtu.be/V8PySHIgXss
Blood Pressure Myths	http://youtu.be/mJ0WpWFURrQ
How to Lower Blood Pressure	http://youtu.be/4lJY1UkqQbs
High Blood Pressure Defined	http://youtu.be/hJklQCNGUvw
Diagnosing High Blood Pressure	http://youtu.be/rkv2w3In4lk
Treating High Blood Pressure	http://youtu.be/aVxRazMKmdo
What is White Coat Syndome?	http://youtu.be/Fkvw1x-A7-4

What is Atrial Fibrillation?	http://youtu.be/CNpX3-b6P_Y
Risk Factors for Women's Heart Disease	http://youtu.be/KB6EdoF99Ag
AFIB: Life Changes	http://youtu.be/ocpvjUmBSQ0
AFIB: Complications	http://youtu.be/P8qiPsWLPBY
AFIB: Risks and Causes	http://youtu.be/5hdS7SPey7U
AFIB: Diagnosis	http://youtu.be/pDtc_UVrGf4
Hormone Therapy and Heart Disease	http://youtu.be/enMWSFVem_g
Heart Disease in Women Number	http://youtu.be/9XENyVj9MUU
Depression and Women's Heart Disease	http://youtu.be/QtxMPw41R58
Pregnancy and Heart Disease	http://youtu.be/qVF8qq9mCS0
Challenges for Women with Heart Disease	http://youtu.be/c3DlCEb2pNs
AFIB:Treatments	http://youtu.be/z2IoIkPUi2w
I Am Ready to Beat My Family's Curse of Heart Disease	http://youtu.be/Z32it3RGD50
Heart Disease: Explaining Dangerous Arrhythmias	http://youtu.be/4bvD1Fau9Ws
Heart Disease: A New Perspective on Life	http://youtu.be/WGjCFgqG8pw
Heart Disease: Staying Positive	http://youtu.be/VtPM8E7Ffk8
Heart Disease: One Family's Struggle	http://youtu.be/_sjCEI_S9lE
Heart Disease: A Life-Changing Diagnosis	http://youtu.be/p06qd98kZxo
Congenital Heart Disease: Sisters Sizzle in Cook-Off	http://youtu.be/XoZkHjtwMcw
Congenital Heart Disease: A Family's Triple Diagnosis	http://youtu.be/B-KcsdoiTaA
Congenital Heart Disease: Heart Healthy Cook-Off	http://youtu.be/xr_UE8NE34o
Heart Disease: Teenage Sisters Living with a Rare Disease	http://youtu.be/MFQJGN2GMAQ
When To Check Your Blood Pressure?	http://youtu.be/hlbhdtx-2ks
Transcatheter Aortic Valve Replacement (TAVR) at University Health System	http://youtu.be/M-iUzl__dSA
Transcatheter Aortic Valve Replacement (TAVR) at University Health System	http://youtu.be/6x3a-uJ03nw
Video Footage of Edwards SAPIEN Transcatheter Heart Valve at Work	http://youtu.be/2chcCKltngA
Edwards SAPIEN Transcatheter Heart Valve Deployment - TAVR	http://youtu.be/FmojTCau6l8
Transfermoral Deployment of Edwards SAPIEN Transcatheter Heart Valve in Calcified Aortic V	http://youtu.be/m1q3n-jKAzk
What's Your Heart Age?	http://youtu.be/NPJ-PFTeamY
Lessons From the Heart	http://youtu.be/b3OjLRChsLA
Cardiology at University Health System - Living Proof Webisode	http://youtu.be/yPjsQrxYCoU

News 4-WOAI: Signs You're Having a Heart Attack	http://youtu.be/g-dZyxaMsXk
Mrs. Munoz on Heart Attacks in Women	http://youtu.be/dbtGf3m1Qjw
Mrs. Munoz on Heart Health	http://youtu.be/YhUO_IZU-eQ
New heart procedure transmyocardial laser revascularization performed at University Hospita	http://youtu.be/s9OmlntS1kg
New Telemetry System	http://youtu.be/hcyK-bOS3DE
Cardiology at University Health System - Living Proof	http://youtu.be/anjkk24LLyo
Brian Jones - Aortic Dissection - Real Patients. Real Stories	http://youtu.be/G8yMsK9gb2I
Spotlight on Women's Heart Health	http://youtu.be/oGSBhpfv50U
Patricia Cochran - Heart Attack - Real Patients. Real Stories	http://youtu.be/526GYXrJ1uk
National Wear Red Day at THE HEART HOSPITAL Baylor Plano	http://youtu.be/FgIlbfpNJlM
Kelli Freeman - Heart Valve Surgery - Real Patients. Real Stories	http://youtu.be/CPpO4Op3Hto
Real Patients. Real Stories. Tricia Kot - Heart and Vascular Health	http://youtu.be/eRkNdfnXWDo
Real Patients. Real Stories. Matt Kieffer - Heart & Vascular Health	http://youtu.be/HtS2yT1LO0M
Ask the Expert: Syncope	http://youtu.be/4n6jxikl2v4
Ask the Expert: Radial Artery Catheterization	http://youtu.be/hSFP6uMg0hY
November 15th Heart Hospital Seminar	http://youtu.be/ugK4mC5Mr08
Transcatheter Aortic Valve Replacement - Dr. David Brown	http://youtu.be/cn-UpJzvtSM
Ask an Expert: Atrial Fibrillation	http://youtu.be/epzmb4S5ySw
Ask the Expert: Atrial Fibrillation	http://youtu.be/VkYIhaKc2ZQ
Real Patients, Real Stories: Alvin Wade Heart & Vascular	http://youtu.be/SQ00B171i8g
Real Patients, Real Stories: Debbie Cassetty Heart & Vascular	http://youtu.be/pUrcbwJfyTM
Real Patients, Real Stories: Tommy Spillar Heart & Vascular	http://youtu.be/cQSBYbw6iYY
Real Patients, Real Stories: Cynthia Fountain Heart & Vascular	http://youtu.be/YQq27Da9ngo
Real Patients, Real Stories: Larry Cherry Heart & Vascular	http://youtu.be/xSHBfqwIm04
Real Patients, Real Stories: Mark Allison Heart & Vascular	http://youtu.be/xUIDwTLLxIU
Real Patients, Real Stories: Chuck Billings Heart & Vascular	http://youtu.be/9ULKOy-hnsQ
Real Patients, Real Stories: Rhonda Revels Heart & Vascular	http://youtu.be/Qz5_opdUZGU
Real Patients, Real Stories: Chris Langston Heart & Vascular	http://youtu.be/JIVbtyjN-Go
Baylor Health Care Real Stories: Fred Curry	http://youtu.be/h7uKBvKhSCg
Chad Wheeler's new heart donated by a 27-year-old soldier	http://youtu.be/PlPfA84lYtI
Darlene Clayton: Baylor Health Care Heart Transplant Patient	http://youtu.be/cAY0yxMV4lg

Baylor Health Care Heart Transplant Story	http://youtu.be/nzzyzh44mc8
Baylor Health Care System: Larry Cherry receives a left ventricular assist device for Christmas	http://youtu.be/o5myt_OVOYc
Baylor Health Care System: CPR Saved Carrie Wilson's Baby's Life	http://youtu.be/Mudiiq3iHAU
Ask the Expert: The Link Between Salt Intake and Heart Disease	http://youtu.be/onWtndwgPBI
Real Patients. Real Stories. Rick Figueroa Dallas Heart Patient.	http://youtu.be/kp8w9h4U2JY
Patricia Cochran: I Don't Remember My Heart Attack, But I'll Never Forget Baylor	http://youtu.be/l0TUWeDY-v0
B.J. Watkins: I Didn't Need a Triple Bypass, I Just Needed Baylor	http://youtu.be/_1wN_mdGi7c
How Eating Breakfast Lowers Cholesterol	http://youtu.be/KsXjZ7hUnrU
Study Shows High Blood Pressure Affects Life Expectancy	http://youtu.be/7L_b0fhyTMY
Why Smokers Have Higher Risk of Heart Disease	http://youtu.be/ugnERAFq7as
Heart Attacks & Depression	http://youtu.be/RND_o8-fbks
Napping Decreases Risk of Heart Attack	http://youtu.be/lwngkaD1jqs
Infections Trigger Heart Attacks	http://youtu.be/Cvvor9C_Jxw
Women at High Risk for Strokes, Not Aware of Signs of Stroke in Women	http://youtu.be/UHYPEDgd8_0
You Can Have a Stroke and Not Recognize the Signs	http://youtu.be/Lj0B3aSOWPQ
Traffic Triggers Heart Attacks	http://youtu.be/HPzSkWzOX7Y
How to Tell the Difference Between Panic Attacks and Heart Attacks	http://youtu.be/oqiA-R06gtU
Silent Heart Attacks: Symptoms You Might Not Recognize	http://youtu.be/fETsQT1X5d0
Women Often Overlook Symptoms of Heart Attack Until It's Too Late	http://youtu.be/TYQZq_Sbero
Heart Disease? Maybe You Can Blame Your Mother	http://youtu.be/kQrqBYn7juA
Benefits of Lowering Cholesterol at a Young Age	http://youtu.be/35K4yzTg_XA
Hypertension, High Blood Pressure, and Why You Need to Check Your Blood Pressure	http://youtu.be/Q6HHVzJfHXg
'Tis the Season for Heart Attacks	http://youtu.be/usxR58I8uR4
CARDIAC CATHETERIZATION	http://youtu.be/WiN776pyEZM
My Doctor My Health - Hypertension	http://youtu.be/R72O7Gdi9pQ
My Doctor My Health - Dislipidemia	http://youtu.be/6B22E4Wvgt8
Are You HeartAware?	http://youtu.be/V37sdisikIk
Shelby Baptist Cardiologist Dr. Dale Elliott	http://youtu.be/ICASs0V2SlE
Princeton Baptist Cardiologist Dr. Michael Wilensky	http://youtu.be/l_pb2Q9SFYo
What is HeartAware?	http://youtu.be/Lv6Z_2tOXOQ
Baptist Health Minute: Women and Heart Disease	http://youtu.be/C-JbUHb8d5k

Baptist Health Minute: Princeton Baptist is an Accredited Chest Pain Center	http://youtu.be/pi2jidxCXQs
Heart attacks occur suddenly	http://youtu.be/J_2VNyhvZYY
What are the Symptoms of Heart Attack? VIDEO HAS BEEN REMOVED	http://youtu.be/avRei6WFm3A
Are You At Risk? VIDEO UNAVAILABLE	http://youtu.be/lzVAHUw72mQ
Video 17 - Heart surgery - A delicate balance of speed and precision	http://youtu.be/YW_TTM9vrdY
Video 19 - A new heart for Rylynn	http://youtu.be/HUdi5yuzrkM
Screening for cardiac problems in young athletes	http://youtu.be/ggM19QIb_2M
Mother's Day Gift Comes in Form of Tiny Heart	http://youtu.be/mw5c7NQj59A
Cardiologist speaks on the dangers of K2	http://youtu.be/Owo-UU_u6Cc
Brooke's Broken Heart	http://youtu.be/9hgqm4TrCX0
Heart transplant patient meets Dallas Cowboys	http://youtu.be/wNSudshRp-U
Pediatric Cardiology at Children's Medical Center	http://youtu.be/qGsKWuPZbdQ
Two sisters on heart transplant list	http://youtu.be/9PwfiOuHrjA
Seventh grader gets new heart	http://youtu.be/Srkc0_VPsPY
Prevent cardiac arrest in athletes	http://youtu.be/anvzzz-pUPo
Hypoplastic Left Heart Syndrome	http://youtu.be/AmutlWI0o7g
Biventricular Repair	http://youtu.be/yC88XfebTAE
Heart problems & sports	http://youtu.be/rGlDwhfI8NI
Heart Attack Survival Story - Tale of Two Eds - Methodist Richardson Medical Center	http://youtu.be/xO-wRN1ptwU
Heart to Heart 2012 -- Women's Heart Health Awareness -- North Texas	http://youtu.be/PxOJGA30g9k
Heart Attack Survivor, New Mom -- Michelle Chambers Methodist Dallas Medical Center Stor	http://youtu.be/QyNr0vEUESA
Heart of the Community -- Methodist Health System North Texas	http://youtu.be/Zpo3Etv5quI
Cardiology and Women and Children's Services at Methodist Health System, North Texas	http://youtu.be/CXxx0zcjlSA
Methodist Mansfield Heart to Heart Community Health Event 2011	http://youtu.be/POV_1-U1lpA
Hear the whole story of Johnnie's heart attack to Cath Lab in under an hour.	http://youtu.be/6MRqMpR3rnE
Methodist's Quality Chest Pain Center Gets Johnnie into the Cath Lab in under an hour	http://youtu.be/IvhyKxH7u6I
Methodist's Quality Rapid heart procedures saves Johnnie's life.	http://youtu.be/5T_vd59PmbY
August Heart Screening at Methodist Stone Oak Hospital	http://youtu.be/_OQwvi_u1y0
Methodist TAVR Program	http://youtu.be/15v5bu6FVgg
One-of-a-Kind Cardio Hospitalist Program	http://youtu.be/UYmNyQLNMnY
Heart Transplant Recipients Train for the Rock 'n Roll Half Marathon	http://youtu.be/fXD7WUKJ8Oo

Meet a two-time Heart Transplant Recipient	http://youtu.be/kiANWddEQtU
One Woman's Heart Story	http://youtu.be/xCNLJlLRjmM
McKinney Medical Center - Pacemaker Tune Up	http://youtu.be/7yC1Smadf44
McKinney Medical Center - Healthy Heart and Your Lifestyle	http://youtu.be/nfyVrrZAk8A
McKinney Medical Center - The Cardiac Electrician	http://youtu.be/F75fD4XlHNA
Medical Center of McKinney Chest Pain Center: 39-year-old Heart Attack Survivor	http://youtu.be/loVzp8kCiQA
Recognize Heart Disease Early Warning Signs	http://youtu.be/gBmOL4htAgI
Don't Wait Too Long. Know Heart Attack Symptoms..flv	http://youtu.be/DaUmPtiUNHM
Medical Minute January 31, 2012	http://youtu.be/jJ27ekLOegw
CHRISTUS Hospital - St Mary	http://www.youtube.com/watch?v=6
Dr. Darrin Letsinger, Cardiac Anesthesiologist	http://youtu.be/tyt8lcUSl5s
Dr. Darrin Letsinger, Cardiac Anesthesiologist	http://www.youtube.com/watch?v=J
CHRISTUS Cabrini Hospital 2011 Cause Sponsor of the American Heart Association's "Go Red f	http://youtu.be/xKvosdtfZVk
Symptoms	http://youtu.be/k1KyrF3NqRM
CHRISTUS Cabrini Hospital Cardiology - Stroke Center	http://www.youtube.com/watch?v=8
Cardiology: Peripheral Vascular Disease (PVD)	http://youtu.be/2qEjLpv1A3A
Tour New Cardiovascular Center at CHRISTUS Santa Rosa Hospital - New Braunfels	http://www.youtube.com/watch?v=t
Lee O'Donnell's Story - Orlando Health Heart Institute	http://youtu.be/mGtzKkc94n4
Orlando Health Heart Institute - New TAVR procedure means new hope for patients	http://youtu.be/lbtS2QUAKiw
TAVR - Transcatheter Aortic Valve Replacement	http://youtu.be/o0dHwYapqro
Orlando Health Heart Institute "Lee's Story" Anomalous Right Coronary Artery	http://youtu.be/hHqkYac-ccw
Orlando Health | Heart Attack Warning Signs | Florida's Blood Centers | Amercan Heart Asso	http://youtu.be/uIS8mMombSk
Orlando Health | Know Your Numbers for Heart Health | Florida's Blood Centers	http://youtu.be/Y_SSgjSxU84
Orlando Health | Cardiovascular Benefits of Donating Blood | Florida's Blood Centers	http://youtu.be/IFAO5f7XXyk
Orlando Health | Cardiovascular Benefits of Donating Blood | Florida's Blood Centers	http://youtu.be/oX1Tj39fym4
Orlando Health "Mia's Story" - Hypoplastic Heart Syndrome - Arnold Palmer Hospital	http://youtu.be/Ba2D37VxeY0
Orlando Health "A Steady Heart" - Thomas' Story - Atrial Fibrillation	http://youtu.be/CWbygzj3lgA
Get to Know the Orlando Health Heart Institute - A New Model of Cardiac Care	http://youtu.be/x30I807_RJk
$35 Healthy Heart Screening from Orlando Health Heart Institute: It's Easy!	http://youtu.be/LKAocLMUhSc
Orlando Health Heart Institute "I Believe in my Heart"	http://youtu.be/nHZQic5IHBM
Orlando Health - 2011 Heartwalk - "Work Your Body Out" (clip)	http://youtu.be/P7IBNwRL6KE

Arnold Palmer Hospital - Hybrid Cath Lab	http://youtu.be/jPPBez1uZrQ
Orlando Health and American Heart Association Go Red For Women	http://www.youtube.com/watch?v=b
Orlando Health Stories - Dr. Einhorn	http://youtu.be/hoqWbbiE4go
MRI Compatible Pacemaker	http://youtu.be/R4a-IrdfOOo
ORMC uses first and only pacemaker system for use during MRI	http://youtu.be/QJ1qiQ7cf-I
Orlando Health - Valentine's Day Heart Questions	http://youtu.be/lMic7DBr1Zk
Heart Healthy Recipes for the Big Game!	http://youtu.be/_bLZIrUqbt8
Orlando Health - Heart Man Returns	http://youtu.be/UtVVQuN3c2s
Cryocath animation	http://youtu.be/J_4E6SP__hE
New Surgical Technique Helps Prevent Stroke	http://youtu.be/TFs8tFoF9Fw
Orlando Health - Heart Healthy Cookout	http://youtu.be/zCgAfCxm394
Orlando Health - South Seminole Hospital - High Blood Pressure	http://youtu.be/mP8PZWe5e24
Orlando Health - Orlando Regional Medical Center - Aortic Valve Replacement	http://youtu.be/OqoC6YZc6cg
Orlando Health - Orlando Regional Medical Center - Pacemakers	http://youtu.be/o9iQtWhBmt4
Orlando Health - South Seminole Hospital - Stroke Treatment	http://youtu.be/44H3lJJv5es
Orlando Health - Dr. P. Phillips Hospital - Chest Pain Center	http://youtu.be/mgIx_jbG2yo
Orlando Health - Arnold Palmer Hospital - Pediatric Cardiology - Heart Murmur	http://youtu.be/vBPfnrVgkdY
Orlando Health - Arnold Palmer Hospital - Pediatric Cardiac Intensive Care Unit	http://youtu.be/oU5AVBkYFHQ
Orlando Health - Arnold Palmer Hospital - Pediatric Cardiac Catheterization	http://youtu.be/zbbDjaaxu50
Orlando Health - Dr. P. Phillips Hospital Stroke Awareness Day Event	http://youtu.be/J1SLzoCXNHo
Orlando Health - Congenital Heart Institute Valentine's Day Celebration 2010	http://youtu.be/FvkOxOY6-y8
Orlando Health - Arnold Palmer Hospital CVICU Open House	http://youtu.be/XPuMEG2jfSI
Orlando Health - Seminole County Regional Chamber of Commerce Moves It for Heart Health	http://youtu.be/cw_eSG-hXB4
Orlando Health - The Latest Heart Valve Breakthroughs [HQ]	http://youtu.be/QeUbCRhdZ98
Orlando Health - Dr. Einhorn Discusses Heart Disease	http://youtu.be/lQIEo0sqJ7o
Orlando Health - Dr. Duran discusses heart arrhythmias. [HQ]	http://youtu.be/uHkVfQFJI48
Orlando Health - Move It For Heart Health	http://youtu.be/5n_Ch5UAXOE
Orlando Health - Dr. Dalton Explains Cardiac Catheterization	http://youtu.be/zFAXZJ6RG9Y
Orlando Health - The only extra beat you'll experience, is on the dance floor!	http://youtu.be/Apz8NL4FLek
Orlando Health - Heart on the Line	http://youtu.be/Z_2U1xZM4qM
Baptist Hospital's heart surgery program achieves highest rating	http://youtu.be/0SbSumbizxo

Abdominal Aortic Aneurysms (AAA)	http://youtu.be/b5up4cBtDR4
What is a Heart Attack?	http://youtu.be/PXnHjJXQpfc
Atrial Fibrillation or irregular heart beat Patient Story, Efrain H. Gonzalez, MD	http://youtu.be/yR-l1yOyIFI
What is Atrial Fibrillation or irregular heart beat?	http://youtu.be/FKJIV6xAQv4
2012 International Cardiovascular Disease Prevention Symposium - Interviews with the Facult	http://youtu.be/7EF41NCfHOo
2012 Coronary CTA in the Emergency Department: A Hands-on Workshop	http://youtu.be/B267pbUt-IQ
Abdominal Aortic Aneurysms (AAA) Patient Testimonial	http://youtu.be/vpeiY_IxgmU
Heart Disease Risk Factors	http://youtu.be/gVvS0lmc4X0
Baptist Cardiac & Vascular Institute Bringing Innovation to Life	http://youtu.be/Yhx9fMA5RYY
Carotid Artery Disease Patient Story by Barry Katzen, MD, and Athanassios Tsoukas, MD	http://youtu.be/EVh8tEIQ2U8
Cardiac Rehab Helps Heal the Heart	http://youtu.be/RNoc1Ws4BCE
Atrial Fibrillation Medical Tip by Baptist Cardiac & Vascular Institute's Ramon Quesada, M.D	http://youtu.be/xtlPjxkJlVc
Atrial Fibrillation Patient Testimonial at Baptist Cardiac & Vascular Institute	http://youtu.be/9ei4WiQzu0c
Baptist Cardiac & Vascular Institute performs its 1,000th abdominal aortic aneurysm	http://youtu.be/rFnaAbFgdcY
Minimally Invasive Aneurysm Treatment & Stroke Warning Signs - Dr. Italo Linfante M.D	http://youtu.be/WLez8cRjxEw
Celine Dion - Baptist Cardiac & Vascular Institute- NBC 6	http://youtu.be/q9EfjJfpA_0
Baptist Cardiac & Vascular Institute	http://youtu.be/53m07nmAHvo
Know Your Numbers -- Theodore Feldman, M.D.	http://youtu.be/eJTt_NZkOXo
How Women's Heart Attack Symptoms Differ from Men's -- Stratego Castanes, M.D.	http://youtu.be/FyOB1Ef68h8
Heart Attack Unit -- Dan Krauthamer, M.D.	http://youtu.be/mZOP3NkOtcY
First MRI Safe Pacemaker Implanted in South Florida Patient	http://youtu.be/BB9DLiNxHpI
Risk Factors that Develop Heart Disease - Harry Aldrich, M.D.	http://youtu.be/E0VTKXK1VcQ
Heart Disease in Women - Abbe Rosenbaum, M.D.	http://youtu.be/tx3iS_0NmRk
Decreasing the Risk of Heart Disease - Dean Heller, M.D.	http://youtu.be/UzOaIIigMEY
Atrial Fibrillation - Efrain Gonzalez, M.D.	http://youtu.be/RcP4pWzvJZA
Pacemaker Defibrillator to Treat Heart Disease	http://youtu.be/tu81b8l7JhQ
Balloon Angioplasty to Treat Blocked Coronary Artery	http://youtu.be/NE_EzcrHbFk
Patent Foramen Ovale (PFO) - Ramon Quesada, M.D.	http://youtu.be/qkh-7UbJ0GM
Health Health Tips - Dr. Rosenbaum	http://youtu.be/1YgBpeyeexo
Health Health Tips - Dr. Aldrich	http://youtu.be/-uamfsI3pkE
Health Health Tips - Dr. Dean Heller	http://youtu.be/pNHnV7fC83c

Health Health Tips - Dr. Efrain Gonzalez	http://youtu.be/SCLprN0-IGo
2011 - Cardiovascular Disease Prevention 9th Annual Symposium	http://youtu.be/Hrbf99zdwhk
The Use of Echocardiography to Diagnose Heart Problems - South Miami Heart Center	http://youtu.be/3Bc38R-VlKk
The Importance of Exercise for Maintaining Cardiovascular Health - South Miami Heart Cente	http://youtu.be/vMfV8h-RPlY
Heart Disease in Women - South Miami Heart Center	http://youtu.be/VKKEdChkZ0M
Treating Symptoms of Heart Failure South Miami Heart Center	http://youtu.be/jEcEEWlceRQ
Diagnosing for Heart Condition Symptoms South Miami Heart Center	http://youtu.be/Ny5zNOVoXos
Cardiac Catheterization and Heart Stents Can Save Lives- South Miami Heart Center	http://youtu.be/CIMAzhlmlGo
Steven Kang, M.D. - What is Peripheral Arterial Disease or PAD?	http://youtu.be/iAac6a_748M
What Happens During a Heart Attack, and What You Can Do South Miami Heart Center	http://youtu.be/FIb457jWZ80
Know Your Numbers to Reduce your Risk for Heart Disease - South Miami Heart Center	http://youtu.be/LRLN5Qa210g
Proper Use of a Portable Automatic External Defibrillator - South Miami Heart Center	http://youtu.be/wRI8T1mEkI0
Why Group Community Education on Heart Health Is Important - South Miami Heart Center	http://youtu.be/s8AbQHDM40A
2010 Cardiovascular Disease Prevention 8th Annual Symposium	http://youtu.be/-8Fs98SeGGw
Brooks Rehabilitation Stroke Recovery	http://youtu.be/vZlaVbVjPjQ
Brooks Heart Healthy Cooking Class: Simple Mediterranean Vegetarian Pasta	http://youtu.be/hpWzbfWna6Y
Brooks Heart Healthy Cooking Class: Sprouted Mung Beans	http://youtu.be/Fd4UKmMvO1Y
Brooks Heart Healthy Cooking Class: Sprouted Hummus	http://youtu.be/FOcbSqGu9Bo
Effects of a Stroke on The Brain, Left vs Right	http://youtu.be/uEPPPXzLMLo
Stroke Prevention - Modifiable Risk Factors	http://youtu.be/xVHrRs5lLTI
Driving After A Stroke in Florida	http://youtu.be/zMx0L1qthGA
The Heart Center at Holmes Regional Medical Center	http://youtu.be/IPtmM4wKq-0
PFO Closure Animation 2	http://youtu.be/N9yX2oLxuyM
Young Stroke Victim Returns to Aventura to Thank Staff	http://youtu.be/pNeml8O0YeA
PFO Stroke Animation	http://youtu.be/V4CnttAApSU
PFO closure animation	http://youtu.be/LfAdmWWKcB0
Women's Heart Disease	http://youtu.be/wNxTvDGQj0A
Peripheral Vascular Disease	http://youtu.be/OHGgso9FPcE
Dr. Augusto Villa speaks about Heart Month	http://youtu.be/tZEZ4meIrjk
Michelle's Heart Story	http://youtu.be/gT_YgNW7Otw
Palm Beach County woman survives heart attack with high tech pump, teamwork	http://youtu.be/UBNUT6sHCWI

Dr. Matthew Klein on new A-Fib Procedure	http://youtu.be/l6ZtBx3jRDw
Dr. Klein on A-Fib Procedure	http://youtu.be/HpH992qUUs8
Heart Institute	http://youtu.be/WtfEZ3Lymd4
Door to Balloon	http://youtu.be/9FOhh28KO-Y
Chef Stephen Bloomer of PBGMC on CBS 12 Morning Show Promoting Heart Healthy Meals 2	http://youtu.be/ZH_sHqnsYdQ
Chef Stephen Bloomer of PBGMC on CBS 12 Morning Show Promoting Heart Healthy Meals	http://youtu.be/bn9RRGFRKQQ
Heart Health Video With Palm Beach Gardens Medical Center Physician Dr. Villa	http://youtu.be/q4kLl5aYRIQ
Heart Health Video with Palm Beach Gardens Medical Center Physician Dr. Klein	http://youtu.be/gREIJf3WaLQ
Heart Health Video with Palm Beach Gardens Medical Center Physician Dr. Faro	http://youtu.be/63IrvWvR2Gc
Heart Health Video with Palm Beach Gardens Medical Center Physician's Dr. Breuer and Dr. K	http://youtu.be/eXDtHJUJvNM
Heart Health	http://youtu.be/pfg35NewjpU
Dr. Nils Mueller Discusses the Use of Solitaire Device	http://youtu.be/XBPrRh_EBws
Cardiac Care	http://youtu.be/M-X_Hy8lsE0
Hypothermic Blanket at Delray Medical Center story on the FOX 10pm Newscast	http://youtu.be/bBxdfJLy30M
Dr. Mueller on new Stroke Medicine	http://youtu.be/8CDNf8OtaLY
Heart Month Health Tips With Cardiac Surgeon	http://youtu.be/lGYkYL_Gg54
Mona Mishalow, Pacemaker Recipient	http://youtu.be/3fVzh6fXzk0
Howard Raymer, Quadruple Bypass	http://youtu.be/Cm8v05E_doY
Mark Bleiweis - Hypoplastic Left Heart Syndrome :60 Commercial	http://youtu.be/esUipOILGVc
Mark Bleiweis - Hypoplastic Left Heart Syndrome :30 Commercial	http://youtu.be/xNdtB9BRvjw
Mark Bleiweis Interview	http://youtu.be/qr9aUYnSfIo
Comprehensive Stroke Center	http://youtu.be/PJOCLe_7vKw
Meet UF Physician Dr. Mark Bleiweis, MD	http://youtu.be/UZJiS5nAzDA
Hypoplastic left heart syndrome	http://youtu.be/SltjHLRtFBk
Ventricular Assist Device (VAD): Shands HealthCare and UF physicians	http://youtu.be/DBqt1hCNZng
About the "Mini Maze" Procedure for Atrial Fibrillation	http://youtu.be/1QUy3Ro1mdM
Memorial Cardiac and Vascular Institute -- One Name to Remember	http://youtu.be/bdcqGfx49ow
TAVR at Memorial Cardiac Vascular Institute	http://youtu.be/BuQ9Tn4JkkQ
If You're Having a Stroke, Would You Recognize the Symptoms?	http://youtu.be/UAaFmBRVFXI
Cardiac Surgeon Saves Baby with Multiple, Life-Threatening Defects	http://youtu.be/lVCx5vm44z8
Memorial Cardiac and Vascular Institute, Michael Cortelli, MD	http://youtu.be/pPCklHXj_yA

Memorial Cardiac and Vascular Institute, Lance Cohen, M	http://youtu.be/a1rFLMHcDvc
Memorial Cardiac and Vascular Institute, Juan Plate, MD	http://youtu.be/Llj_lJbJFuk
Memorial Cardiac and Vascular Institute, Richard Perryman, MD	http://youtu.be/9EvZ61wtTxs
Follow Your Heart to Memorial	http://youtu.be/VeZwm377Jb0
Girl's Miracle Stroke Recovery at Joe DiMaggio Children's Hospital	http://youtu.be/2qlRCLVZoAI
Heart Disease Is An Epidemic	http://youtu.be/jWZ2mfECKp8
Heart Disease in Women	http://youtu.be/zPtLtHVHojo
Treating Congenital Heart Defects at Joe DiMaggio Children's Hospital	http://youtu.be/nCN5kCPvdJM
What is Congential Heart Defect?	http://youtu.be/lqRRHoKY0qg
Heart Attack: Know the Signs	http://youtu.be/ZcOwpiZQ3vY
Memorial Helps Put Young Stroke Patient Back in the Running	http://youtu.be/wC7wu-9czo4
The Cardiac Center at Joe DiMaggio Children's Hospital	http://youtu.be/28A9TW3Lnl0
Cardiac Surgeon Saves Baby With Multiple, Life-Threatening Heart Defects	http://youtu.be/Hs7A_79IfNk
From Patient to Caregiver, Katie's Giving Back from the Heart	http://youtu.be/oKlpPOwTPZE
First Pediatric Heart Transplant at Joe DiMaggio Children's Hospital	http://youtu.be/g2RT7E12rnU
Cardiac Patient Bypasses His Fears, Thanks to His Memorial Doctor	http://youtu.be/tMtEBDdQEyk
Women & Hypertension	http://youtu.be/UCrSecyvvLw
Tuning into A-Fib	http://youtu.be/1Pc8-pfnpH8
Cutting Edge Blood Clot Removal	http://youtu.be/8w6qZsDQ4aQ
Women in a Heart Beat	http://youtu.be/HAwBxr-Cb-s
What to Expect From Cardiac Rehab	http://youtu.be/uZBwqt3UlG4
Keeping your Heart Pumping	http://youtu.be/LJhbfuRHmB8
Brain Attack- Breaking Down Stroke	http://youtu.be/iyNpQvmzAlI
Apnea & Heart Risk	http://youtu.be/lXMQjxcE2DM
The Aging Heart	http://youtu.be/MY373mYi3ww
Diabetes & Heart Disease	http://youtu.be/rm2rvRc168s
Stent & Angioplasty- It's all in the Wrist	http://youtu.be/bmvPHRmnBbw
Unrecognized Heart Attacks	http://youtu.be/0Kfj0Y9DkW8
Working the Heart Muscle	http://youtu.be/J-07FlZJP-E
Cardiac Wakeup Call	http://youtu.be/CznaAVF613Y
The Failing Heart: Breaking Down Congestive Heart Failure	http://youtu.be/xWSTtdZWm3U

Scanning for Strokes	http://youtu.be/YrpWJfMIt-Q
Have you Checked your Cholesterol Lately?	http://youtu.be/oAWnbKETlh4
Simplifying Heart Surgery	http://youtu.be/yDRrX2ykTpY
Taking Steps to Treat PAD	http://youtu.be/EdRkAaCCHKg
Lady Killers: Hidden Heart Disease	http://youtu.be/bpZB_h3FOyA
Saving Hearts with LVAD	http://youtu.be/Mw3A1hc510I
Food for The Heart	http://youtu.be/RZlBGh1HTEA
Life Preserver for the Heart	http://youtu.be/c0ORpFpxzDE
Why BMI Is Important To Your Heart	http://youtu.be/62DRJ-IIkAs
A History of Heart Disease	http://youtu.be/_h4v4vjCFSk
Getting Stroke Patients Back on Their Feet	http://youtu.be/cCCSs9aXpDk
Hints You May Have Heart Disease	http://youtu.be/EzHP0yGaXy8
Heart Attack Myth Busters	http://youtu.be/DPITRynfv_s
Congestive Heart Failure Fact and Fiction	http://youtu.be/YRG-K1hB6rA
Keys to Drug Coated Heart Stents	http://youtu.be/rJPnhDJfSPs

New Ways to Save Hearts Angina http://youtu.be/[illegible]
Salt and Stroke Risk Stroke http://youtu.be/U-1DQ6bvfKA
Reading your Heart's Warning Signs Angina http://youtu.be/zWmpTZQjXHc
Stressless Stress Tests None http://youtu.be/_4lJ6GzHAhs
Speech After Stroke-Treating Aphasia Stroke http://youtu.be/brzAuuOuvqA
A New Path for Cardiac Catheterization Angina http://youtu.be/7azaJlxDutY
Aortic Stenosis: The Aging Heart Disease Valvular Disease http://youtu.be/-oYMbDLYaGE
Living with Congestive Heart Failure Heart Failure http://youtu.be/G_bCWHaZ0bg
Top Tips to Avoid a Stroke Stroke http://youtu.be/FTfuVsYwbNQ
Wearable Defibrillators Dysrhythmia http://youtu.be/iYpi2OLuqOM
Regaining Control with Stroke Therapy Stroke http://youtu.be/FfXeRKhtxRU
New Solution for Age Old Heart Condition Valvular Disease http://youtu.be/o36o3AY6QtQ
Taking Care of the Female Heart Angina http://youtu.be/ZK-20CzNh4k
Treating PVD the Endovascular Way Peripheral Vascular
Disease http://youtu.be/68q3FlFSMEY
TAVR Transcatheter Aortic Valve Replacement Valvular
Disease http://youtu.be/vVR54BnfFFc
Stroke Prevention Stroke http://youtu.be/KCuN2xVkfX0
Congestive Heart Failure Diagnosis and Treatment Heart
Failure http://youtu.be/cVrp3EtyOcI
Defibrillator-Pacemaker: What's the Difference? Dysrhythmia http://youtu.be/3il0ii7Svwk
Breakthrough Procedure for Heart Valve Surgery Valvular
Disease http://youtu.be/M5diF9Y6YN4
Heart Procedures Performed Through the Wrist Angina http://youtu.be/Im8HIePKwvE
Hypertension, Pre-hypertension & Stroke Stroke http://youtu.be/U-zQsomMWVM
Keeping your Arteries Open with a Stent Angina http://youtu.be/jUudDON8igI
Mini Stroke can Shorten Life Expectancy Stroke http://youtu.be/LalQpRAbj_o
Stem Cells May Repair Broken Hearts Angina http://youtu.be/QoOe477nyjs

[illegible]NyLIYK6spE

[illegible]DT9mihEhRA

[illegible]4F7Q2HKBTo

[illegible]upmWPMWMl8

ɔRROrpVB6NA
